# Results of a non-randomized, open-label phase I study evaluating the novel immunomodulatory peptide TCP-25 for treatment of dystrophic epidermolysis bullosa

**DOI:** 10.1186/s13023-025-04156-7

**Published:** 2025-12-02

**Authors:** Karl Wallblom, Katja Holmgren, Sigrid Lundgren, Emma Belfrage, Torborg Hoppe, Matilda Hugerth, Anna-Karin Lindqvist, Enikö Sonkoly, Artur Schmidtchen

**Affiliations:** 1 Division of Dermatology and Venereology, Department of Clinical Sciences Lund, Lund University, Lund, Sweden; 2 Department of Dermatology, Skane University Hospital, Lund, Sweden; 3 Section for Dermatology and Venereology, Department of Medical Sciences, Uppsala University, Uppsala, Sweden; 4 Xinnate AB, Lund, Sweden

**Keywords:** Epidermolysis bullosa (EB), TCP-25, Immunomodulatory peptide, Wound healing, Anti-inflammatory, Antimicrobial, Clinical trial, Safety study

## Abstract

**Background:**

Despite the high morbidity and severe consequences of epidermolysis bullosa (EB), current treatments remain inadequate, lacking efficacy and the ability to target the microbial and inflammatory components of EB wounds. In this first-in-human open-label, single-arm study—part 3 of a 3-part Phase 1 clinical trial—we examined the safety, tolerability, and systemic exposure of topically applied TCP-25, a thrombin-derived C-terminal peptide with demonstrated anti-inflammatory, antimicrobial, and wound-healing properties in murine and porcine infection models.

**Results:**

Five patients with inherited recessive dystrophic EB received TCP-25 gel, applied to a 50-cm^2^ primary wound treatment area, which was compared with a reference wound treatment area which received standard wound care alone. TCP-25 gel was also applied to a 50-cm^2^ secondary wound treatment area of a higher age or in a more complicated area. TCP-25 gel was administered at escalating doses of 7.25 mg, 14.5 mg, 21.5 mg, and 43.0 mg, 3 times per week over 4 consecutive 1-week treatment periods. For the primary safety endpoint, 11 adverse events were recorded, 10 unlikely related to treatment and mild or moderate in severity, with one possibly linked to gel adhesion. Additionally, no abnormal local reactions were observed in any wound treated with TCP-25, nor in any reference wound, compared to expected wound healing outcomes. For the secondary endpoint, we did not observe any systemic plasma uptake of TCP-25 in any participant. In an exploratory analysis, we observed a steady reduction in open wound size in TCP-25-treated primary wounds, culminating in a median reduction of 76% from baseline to Day 29. Reference wounds exhibited variable changes in wound size, including some increases in size at earlier time points. Secondary wounds that were treated with TCP-25 also decreased steadily, reaching a median open wound size reduction of 78% on day 29. Additionally, TCP-25 demonstrated potential efficacy in accelerating wound healing.

**Conclusions:**

Topical TCP-25 gel was safe and well tolerated and showed no systemic uptake in patients with dystrophic EB, demonstrating promising effects on wound healing.

**Trial Registration:**

Clinical trial NCT05378997 was registered 2022–05-12 (https://clinicaltrials.gov/study/NCT05378997) and amended 2024–02-12 to include Part-3 on dystrophic EB. The amendment was prospectively registered at EudraCT (2021-004728-14).

**Supplementary information:**

The online version contains supplementary material available at 10.1186/s13023-025-04156-7.

## Background

Epidermolysis bullosa (EB) comprises a group of genetic disorders that manifest as mechanically induced blistering and fragility of the skin and other stratified epithelia [1–3]. Consequently, EB is linked to a high wound burden [2]. Subgroups of EB—such as junctional EB (JEB), dystrophic EB (DEB), Kindler’s EB as well as severe forms of EB simplex—are conditions associated with a significant morbidity which severely impacts patients’ day-to-day functioning [4]. For example, DEB can lead to premature death due to complications, including bacterial infection, sepsis [5, 6], and skin cancer [1, 7, 8].

EB wounds can exhibit high levels of proinflammatory cytokines [9], aberrant cytokine-cytokine interactions [10], and altered Toll-like receptor (TLR) and JAK-STAT signaling [10]. These molecular changes are accompanied by significant inflammatory cell infiltration [1], impaired macrophage response and activation [11], and a high content of activated neutrophils [12]. As a result, this combination of excess, chronic, and unbalanced inflammation impairs wound healing and leads to further tissue damage [1] and risk of skin cancer formation [7, 13].

Wounds in EB tend to harbor an altered microbiome, marked by lower microbial diversity and increased colonization by potentially pathogenic species, including *Staphylococcus aureus* and *Pseudomonas aeruginosa*, which in turn increase the risk of infection [14, 15–19]. In infected and hypercolonized wounds, bacteria release proinflammatory products, such as lipopolysaccharide (LPS), that activate TLR signaling and induce excessive, uncontrolled inflammation with high proinflammatory cytokine levels, causing significant proteolysis and thus delaying healing [20].

The current standard of care for treating EB involves wound management using wound care techniques per consensus guidelines [21, 22]. Although advances have been made in these techniques [23], many of the currently recommended treatments—such as silver-containing dressings, creams, and antibiotic ointments—present significant challenges due to issues like complexity of use, potential toxicity, and limited effectiveness [21, 24, 25]. Birch bark extract (Filsuvez) has been approved recently in the EU for topical use in wounds that are associated with DEB and JEB to accelerate wound healing [26]; however, it has demonstrated modest efficacy, with no effects on preventing complications, such as infection, ulceration, and malignant transformation. Targeting the underlying genetic cause, the US FDA recently approved Vyjuvek (beremagene geperpavec-svdt), a topical gene therapy for the treatment of wounds in patients with DEB who all bear mutations in the collagen type VII alpha 1 chain (COL7A1) gene. However, no currently available product collectively targets inflammation, bacteria, and their proinflammatory products, in EB wounds. Thus, these shortcomings have necessitated strategies that encourage faster wound healing and control inflammation and bacteria to improve the symptoms of EB and mitigate the severe consequences of EB wounds.

To this end, thrombin-derived C-terminal peptides (TCPs) constitute a group of endogenous anti-endotoxic and antimicrobial peptides that are present naturally in human wounds [27–29]. The synthetic peptide TCP-25 (GKYGFYTHVFRLKKWIQKVIDQFGE, molecular weight 3088.6 Da), based on the 25 C-terminal amino acids of human thrombin, encompasses several endogenous TCP sequences [29]. TCP-25 can be cleaved into biologically active peptide fragments similar to those naturally generated in wounds in vivo [29]. Functionally, TCP-25 possesses anti-inflammatory activity, neutralizes endotoxins and other microbial products, such as lipoteichoic acid (LTA) and peptidoglycan, and binds specifically to the receptor CD14 to prevent its activation and downstream TLR-mediated inflammatory signaling [30]. In addition to its anti-inflammatory properties, TCP-25 has direct antimicrobial properties, acting against common bacterial pathogens, including *Staphylococcus aureus* and *Pseudomonas aeruginosa* [27, 29], which are major causes of dysbiosis and infection in EB wounds [14, 15–19]. In vitro studies have demonstrated that the antibacterial effects of TCP-25 depend on direct interactions between the peptide and bacteria, resulting in membrane disintegration, permeabilization and bacterial killing [27]. Further, TCP-25 is effective against *Candida* species, inhibiting both fungal growth and the proinflammatory activity of yeast-derived zymosan [31], an important consideration for EB patients, given the frequent occurrence of *Candida* in DEB wounds [15]. Notably, TCP-25 also mitigates excessive neutrophil activation and recruitment—key drivers of inflammation in EB [29, 32].

Specifically with regard to wounds, a TCP-25-containing hydrogel has been shown to reduce both bacterial levels and proinflammatory cytokines in *S. aureus*-infected and *P. aeruginosa*-infected porcine partial-thickness wounds, improving wound healing [29]. Thus, given this efficacy and its combination of anti-inflammatory and antimicrobial properties, based on endogenous innate immune mechanisms, TCP-25 is being developed as a novel treatment modality for EB wounds, for which it was awarded orphan drug designation in 2024 [33]. TCP-25 gel has been applied to human wounds in a 3-part first-in-human clinical trial (ClinicalTrials.gov NCT05378997); this study describes Part 3 of this trial.

The objectives of this study were to evaluate the safety, tolerability, and systemic exposure of 4 dose levels of TCP-25 applied topically to wounds in patients with DEB. In this open-label, single-arm clinical trial, we administered ascending doses of TCP-25 to wounds in patients with recessive DEB (RDEB) and compared them with reference wounds that received only standard wound care on the same patients. We additionally applied TCP-25 gel to a secondary wound on each patient, which was judged by the investigator as more complex due to its location and chronicity.

## Methods

This manuscript was prepared following the CONSORT reporting guidelines and checklist, incorporating the extensions for dose finding, and safety studies [34–36]. As this study was not randomized, checklist items not applicable were omitted.

### Trial design

This open-label, single-arm, ascending-dose study evaluated the safety, tolerability, and systemic exposure of multiple administrations of 4 dose levels of TCP-25. The trial has been registered at ClinicalTrials.gov (identifier NCT05378997) and at EudraCT (identifier 2021-004728-14).

### Participants

This study was conducted in the unit for EB patients at the Dermatology Clinic of Skåne University Hospital Lund, Sweden and at Clinical Trial Consultants ABs Oscar Unit in Uppsala, Sweden from November 2023 to March 2024. Participants were recruited from the dermatology clinics at Skåne University Hospital Lund, Sweden and Uppsala University Hospital, Sweden.

The planned cohort size was 5 participants, and the study was performed in accordance with the European Medicines Agency’s regulatory requirements and following general Phase I safety guidelines. No formal power calculations were performed as the study was dimensioned for primary safety endpoints, with other parameters designated as exploratory outcomes. This trial represented Part 3 of a three-part Phase I study (NCT05378997), enabling progression to the vulnerable DEB population.

The study allowed for inclusion of male or female patients, aged ≥ 15 years, with a documented diagnosis of inherited DEB. Patients had to have at least 3 suitable target wound treatment areas fulfilling the following wound selection criteria as judged by the investigator:

- Including an open wound with a surface area of ≤ 30 cm2
- At Visit 2 (first treatment), each target wound was not to present a surface reduction ≥ 30% from the Screening visit.
- Wound aged ≥ 3 weeks to < 9 months at the Screening visit.

Following these criteria, three 50-cm^2^ wound treatment areas were defined for each participant, as described in Table 1, including a primary wound with a matched reference wound, and a secondary wound with no matching control. The risk of accidental trauma was assessed by the investigator on a wound-by-wound basis. The wound treatment areas included the target wound and the periwound area beyond the border of the open wound to a total treatment area of 50 cm^2^.

**Table 1:** Wound treatment areas

Wound treatment area	Criteria	Assigned treatment
Primary	Not located at an anatomical site with a high likelihood of accidental trauma	Ascending doses of TCP-25 gel
Secondary	Located at an anatomical site with a high likelihood of accidental trauma, OR being within the higher allowed age span	Ascending doses of TCP-25 gel
Reference	Not located at an anatomical site with a high likelihood of accidental trauma Matching the primary wound as much as possible, acts as a control for the primary wound only	Standard wound care (defined below)

Patients with infections that required systemic antibiotics, patients who required systemic corticosteroids, and patients who had undergone stem cell or gene therapy on the target wounds were excluded from the study. The full list of inclusion and exclusion criteria is detailed in the Supplementary Material.

### Interventions

Primary and secondary wound treatment areas received TCP-25 treatment plus standard wound care, defined below. TCP-25—formulated in a proprietary hydrogel based on hydroxyethyl cellulose, containing glycerol for isotonicity and at a pH of 7.0—was administered at the concentrations and volumes in Table 2. The TCP-25 gel was manufactured under GMP as a sterile investigational medicinal product by a licensed contract manufacturer and was supplied by Xinnate AB. Reference wound treatment areas received standard wound care only and were handled in an identical manner to the TCP-25 treated wound treatment areas, with the only difference being that they did not receive TCP-25 gel.

**Table 2:** Dose levels of TCP-25 gel

Dose level	Treatment period (days administered)	**Volume administered (mL/cm**^**2**^)	TCP-25 concentration (mg/mL)	**Dose per cm**^**2**^ **(mg/cm**^**2**^)	Dose per wound treatment area (mg)
1	1 (Days 1, 3, and 6)	0.05	2.9	0.145	7.25
2	2 (Days 8, 10, and 13)	0.1	2.9	0.290	14.5
3	3 (Days 15, 17, and 20)	0.05	8.6	0.430	21.5
4	4 (Days 22, 24, and 27)	0.1	8.6	0.860	43.0

Standard wound care was defined as the regular wound treatment of DEB at the clinics and consistent with expert recommendations [22], but the choice of dressing was limited to silicone-based polyurethane wound dressings, such as Mepilex Lite. Wound areas were cleaned gently per established routines for EB wounds during all dressing changes. Solutions such as potassium permanganate or other local antiseptics were to be avoided during the study for the wound areas included. However, antiseptics could still be used on other wounds not treated with TCP-25, including the reference wound, if clinically mandated based on the investigator’s judgment. Additionally, hypochlorite or potassium permanganate baths could be used if part of the patient’s regular cleaning routine, in accordance with their treating physician’s recommendation. Participants were also allowed to continue standard background treatment, defined as all other treatments they were receiving outside of the protocol, throughout the study. This included both systemic and local concomitant treatments, but excluded systemic anticoagulants and/or corticosteroids at a dose higher than 10 mg prednisolone equivalents daily. To the extent possible, the use of standard background treatments was to remain constant during the study.

Before TCP-25 gel was applied, the wound treatment areas were cleaned according to standard wound care. For these wound treatment areas, the indicated volume of TCP-25 gel was administered using a 3-mL application syringe and then spread using a clean or gloved hand until it covered the entire wound treatment area, which was then covered by a 50-cm^2^ piece of sterile non-adhesive polyurethane wound dressing, such as Mepilex Lite. Alternatively, the gel could instead be applied directly to the 50-cm^2^ dressing and smeared with a clean or gloved hand to encompass the entire dressing before being placed over the wound treatment area.

The dressings were then finally fixed in place using any dressing material that is routinely used for DEB patients. The patients could, based on their regular routines, decide whether they preferred to have either two days between dose 1 and 2 and three days between dose 2 and 3, or three days between dose 1 and 2 and two days between dose 2 and 3. During the study, the same patient followed the same schedule throughout. Additional dressing changes outside of the above were permitted if mandated for wound care, provided study personnel were informed. Wound dressings for reference wounds were changed at the same time as the wound dressings for primary and secondary wounds and cleaned per standard care procedures but did not receive any TCP-25 gel.

### Dose levels and treatment schedule

Four dose levels of TCP-25 gel were administered to 2 wound treatment areas on each participant by topical application per Table 2. We applied a dose escalation strategy in which each dose level was administered 3 times over a 1-week treatment period before progression to the next dose level in each participant. Thus, the 4 dose levels of TCP-25 gel were applied over 4 consecutive treatment periods of 1 week each.

On the first day of each respective treatment period (Days 1, 8, 15, and 22), TCP-25 gel was applied at the assigned dose level to the primary and secondary wound treatment areas by study personnel at the research clinic, and the participants were monitored by the clinical staff for at least 1 hour after application. The dressing on the reference wound area was also changed at this time, and the reference wound area was cleaned according to the standard wound care.

**Fig. 1:**
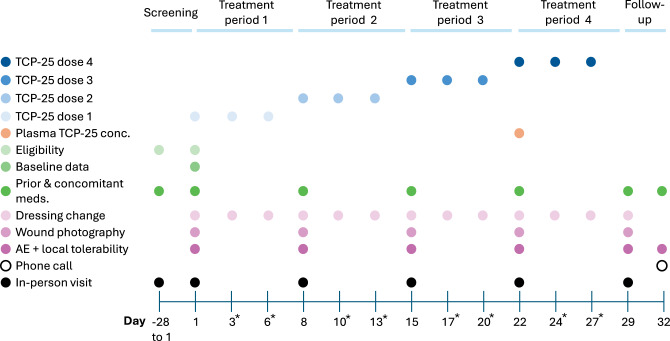
Schedule of treatment and assessments. In each patient, IMP was applied 3 times per week, in total up to 12 doses of TCP-25 gel. *treatment was administered at home by the participant two times a week, preferably on these days. Home administration on the days within brackets was also allowed, if more convenient for the study participants: days 3 (or 4), (5 or) 6, 10 (or 11), (12 or) 13, 17 (or 18), (19 or) 20, 24 (or 25), and (26 or) 27

The second and third dose administration in each week was administered at home by the participant or the caregiver. Study participants were asked to change dressings; clean the primary, secondary, and reference wound treatment areas using standard procedures; and then apply TCP-25 gel to the primary and secondary wound treatment areas per the supplied instructions. They were asked to return all vials of TCP-25 to the research clinic at the next study visit for a compliance check. Participants visited the clinic on Day 29 for a follow-up visit and partook in an end-of-study phone call on Day 32. The dose escalation and assessment schedule is shown in Fig. 1 and Table 2.

At each weekly visit, the investigator evaluated the safety of and tolerability to TCP-25 gel. The participant proceeded to the next treatment period and dose level only if TCP-25 gel at the preceding dose level was deemed safe and tolerable by the investigator.

### Endpoints

*Primary endpoints* - The first primary endpoint was the frequency, intensity, and seriousness of adverse events (AEs). AEs included local tolerability parameters (defined below), infections, and unexpected and excessive hemorrhage. Coagulated blood in wounds was not considered an AE. AEs were assessed during each study visit, on Days 1, 8, 15, 22, 29, and 32 as follows: reported spontaneously by the subject; observed by the investigator or medical personnel; or elicited, based on nonleading questions by the investigator or medical personnel.

AEs were classified by the investigator into categories as defined in CTCAE v5.0 [37]. AEs could be further classified as a serious adverse event (SAE), adverse reaction (AR), serious adverse reaction (SAR), or suspected unexpected serious adverse reaction (SUSAR) by the investigator, the sponsor, or a designee.

A description of each AE was recorded, along with a diagnosis or signs and symptoms, start and stop dates and times, intensity, causal relationship to the treatment or study procedures, actions taken, and outcomes. The intensity of each AE and its causal relationship with the treatment was assessed by the investigator and graded per CTCAE v5.0 [37] and this evaluation. Definitions of causality are specified in the Supplementary Material.

The second primary endpoint was local tolerability. Local tolerability was defined as the incidence of the following abnormal local reactions, compared with baseline, as determined by the investigator on Days 1, 8, 15, 22, and 29. The presence of the following reactions was assessed:

1. Periwound and open wound erythema of the treated area
2. Periwound and open wound edema and swelling of the treated area
3. Periwound and open wound scaling of the treated area

Any abnormal local reactions that were reported here were also recorded as AEs, as described above.

*Secondary endpoint* - The secondary endpoint was the plasma concentration of TCP-25 on the day of the last administration of the investigational medicinal product (Day 22). A single blood sample (2 ×5 mL) was taken 1 hour ±30 minutes postdose on Day 22 (Visit 5). The sample was collected in a lithium-heparin tube and, within 30 minutes of collection, centrifuged at 1500 × g for 10 minutes. The separated plasma from each sample was divided into 2 aliquots in polypropylene cryotubes and frozen at < −70 °C within 1 hour after centrifugation. At the end of the study, all samples were analyzed by Q&Q Laboratories AB, Göteborg, Sweden, using a validated LC-MS/MS method to detect TCP-25, with a lower limit of quantification of 90 nmol/L.

*Exploratory endpoints* - Wound status, evaluated in person at the study visits and based on photographs, was assessed by measurement of the open wound area. To measure open wound area, the primary, secondary and reference wounds were photographed, after removal of the dressing, using a Silhouette wound camera system at the time of the screening and on Days 1, 8, 15, 22, and 29. The wounds were photographed after being cleaned but prior to the application of TCP-25 gel. At least 2 images were taken of each wound. The open wound area was then determined using the Silhouette Connect program.

### Randomization and blinding

Because this trial was an open-label study, no randomization was applied, and the participants, caregivers, investigators, clinical staff who cared for the participants, and all endpoint assessors were not blinded.

### Statistical methods

Continuous data are expressed in terms of evaluable observations, median, minimum, and maximum. Categorical data are presented as counts and percentages. Baseline was defined as the last non-missing observation prior to the first administration of TCP-25. All descriptive summaries were performed in SAS version 9.4 (SAS Institute, Inc., Cary, NC). Descriptive statistics for demographics are presented as median and range for continuous variables and as number of participants, number of events, and percentage of the total for binary variables.

Medical/surgical history is presented by system organ class and preferred term. Prior/concomitant medications are presented by WHO Drug preferred name by treatment and dose group (if applicable) and overall. Medical/surgical history was coded using the Medical Dictionary for Regulatory Activities (MedDRA) version 26.0. Medications were coded using the WHO Drug Dictionary (WHODD) SEP2023.

The incidence of AEs is summarized by system organ class and preferred term by treatment period and overall. Because there was no measurable systemic exposure to TCP-25, statistical analyses of pharmacokinetic variables were not performed.

Summary statistics for open wound size are presented as median and range. Data are presented as absolute size and percentage of baseline size. Open wound size in primary, secondary, and reference wounds on days 1 and 29, as well as the relative reduction in open wound size (baseline compared to day 29), were compared by the Friedman test with Dunn’s correction for multiple comparisons.

The analyses were based on the intention-to-treat population, which includes all enrolled participants. In the case of missing data, no imputation was performed.

## Results

### Participant flow, demographics, and baseline characteristics

**Fig. 2:**
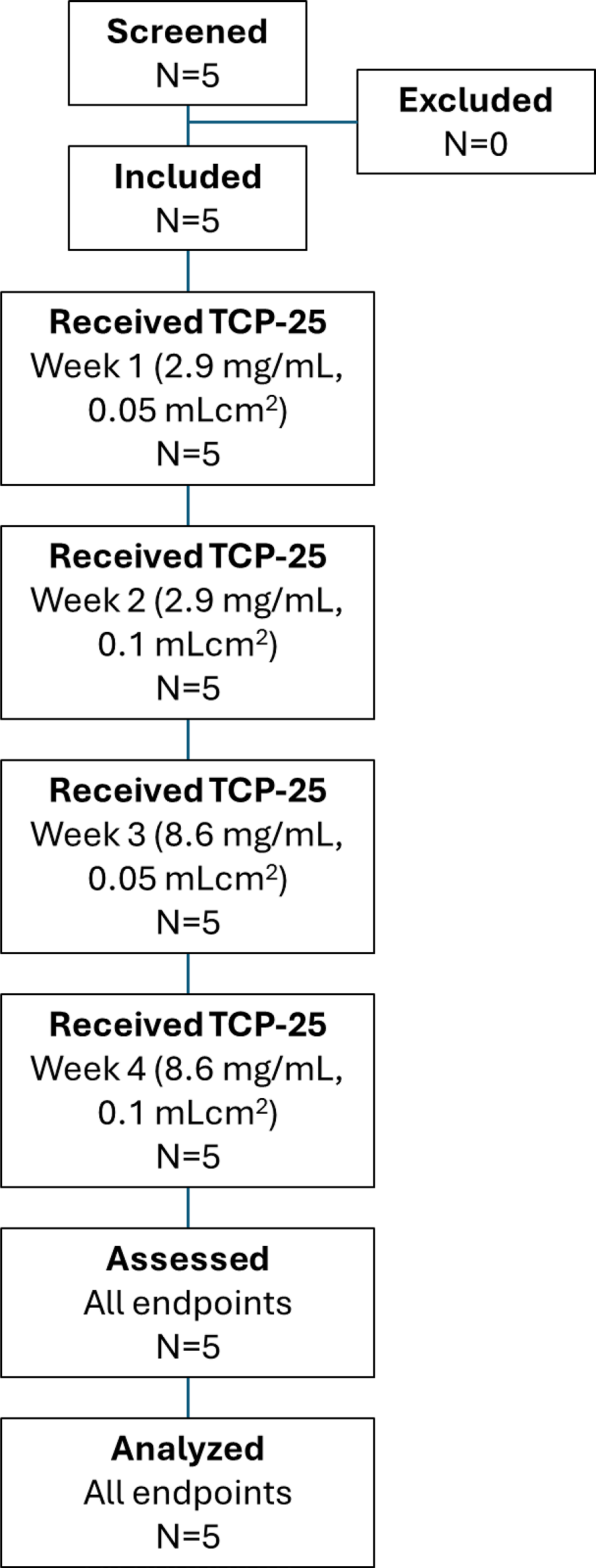
Patient flow diagram with number of patients screened, included, dosed and analyzed in the study

A total of 5 patients with DEB were screened; all 5 were ultimately included in this study and received all 4 dose levels of the assigned treatment (Fig. 2). All 5 participants completed the study. The first participant who was enrolled in Part 3 of this clinical trial was screened on November 29, 2023; the first participant was included on December 6, 2023; and the last participant’s last visit occurred on March 16, 2024.

The baseline characteristics of the participants and their wound treatment areas are described in Tables 3 and 4, respectively. All included patients had confirmed pathogenic COL7A1 mutations, consistent with the diagnosis of RDEB. Detailed genetic information is not reported to protect patient confidentiality. Based on confirmed COL7A1 mutations and clinical phenotype, all participants were classified as severe RDEB. For participants with previously undescribed mutations, subtype classification was determined through clinical assessment by their treating physicians. Collagen VII expression levels assessed by immunohistochemistry showed strongly reduced expression in participant 1, reduced but not negative expression in participant 4, and strongly reduced to negative expression in participant 5. Data on expression levels were not available for participants 2 and 3.

**Table 3:** Baseline characteristics of participants

*Variable*	*Total (N = 5)*	
**Demographics**		
Age; median (range)	18 (15-21)	
Males; n (%)	5 (100%)	
Ethnicity		
Not Hispanic or Latino; n (%)	5 (100%)	
Race		
White; n (%)	1 (20%)	
Asian; n (%)	4 (80%)	
**Medical history**		
Years since EB diagnosis; median (range)	19 (16-22)	
*Previous surgical and medical procedures*	*Number of participants; n (%)*	*Number of events; m*
Gastrostomy	4 (80%)	4
Limb operation	1 (20%)	1
Esophageal dilatation	3 (60%)	3
**Concomitant medications**		
*Most common medications*	*Number of participants; n (%)*	*Number of events; m*
Zinc sulphate (oral)	5 (100%)	5
Betamethasone (topical)	4 (80%)	7
Betamethasone valerate	3 (60%)	4
Betamethasone with neomycin	3 (60%)	3
Alimemazine tartrate (oral)	4 (80%)	4
Carbomer (ophthalmic)	4 (80%)	4
Paracetamol (oral)	3 (60%)	4
Zinc products (topical)	3 (60%)	3
Propylene glycol (topical)	3 (60%)	3
Ibuprofen (oral)	3 (60%)	3
Cholecalciferol (oral)	3 (60%)	3

**Table 4:** Baseline characteristics of the wound treatment areas

Participant	Wound treatment area	**Open wound area at baseline (cm**^**2**^)	Approximate wound age* (weeks)	Wound location
1	Primary	5	5	Upper leg, right
	Reference	2.3	5	Upper leg, left
	Secondary	6	5	Knee, right
2	Primary	6.9	18	Upper leg, left
	Reference	5.8	18	Upper leg, right
	Secondary	17	18	Upper leg, left
3	Primary	1.8	5	Lower leg, left
	Reference	2.4	5	Lower leg, left
	Secondary	17.8	4	Lower leg, left
4	Primary	1.9	8	Hip, left
	Reference	2.3	4	Hip, right
	Secondary	9.6	8	Hip, left
5	Primary	25.5	5	Upper arm, right
	Reference	8	8	Upper leg, right
	Secondary	26.6	8	Upper leg, left

*Wound age was self-reported by participants and thus is only approximate

The included participants were all male and had a median age of 18 years (range 15–21 years). All 5 participants were diagnosed with RDEB at an early age and used concomitant medications for their DEB. None of these topical concomitant medications (see Table 3) was applied to any of the wounds that were evaluated in this study. However, per protocol, potassium permanganate and hypochlorite could be used on non-TCP-25-treated wounds or as a bath if part of the participant’s standard routines, as prescribed by their treating physician. 1 participant reported using chlorine baths, 1 participant reported potassium permanganate baths, and 1 patient reported local potassium permanganate application on non-study wounds. The full list of reported concomitant medications is detailed in the Supplementary Table 1. No use of prior or concomitant medications was judged by investigators to have interfered with the evaluation of the study endpoints. Treatment compliance is detailed in the Supplementary Material.

The open wound areas of the primary and reference wounds at baseline (Day 1) were a median of 5 cm^2^ (range 1.8–25.5 cm^2^) and 2.4 cm^2^ (2.3–8 cm^2^), respectively. The secondary wounds had a median open wound area of 17.0 cm^2^ (6–26.5 cm^2^) at baseline (Table 4).

### Adverse events

All 5 treated participants reported at least 1 AE; a total of 11 AEs were reported for the group overall. There were no deaths, SAEs, other significant AEs, or withdrawals due to AEs. Ten of the 11 AEs were assessed as having been unlikely related to treatment with TCP-25, and 1 event, product adhesion issue, was assessed as possibly related to the treatment. Two participants reported wound-related pain and itching adverse events of mild to moderate severity, affecting both TCP-25-treated and reference wounds, with all events assessed as unlikely related to treatment. All AEs were mild or moderate in intensity. Details on AEs are described in Supplementary Tables 2–4.

### Local tolerability

There were no abnormal local reactions (i.e., erythema, edema, or scaling) at any time, compared with expected wound healing outcomes, in any wound treatment area to which TCP-25 was applied or in any reference wound area, as assessed by the investigator (0 events in all groups).

### Secondary endpoint

TCP-25 plasma concentrations were measured 1 hour ±30 minutes after the last administration of TCP-25 gel to both primary and secondary wound areas (Day 22). However, TCP-25 levels were below the lower limit of quantification (90 nmol/L) in plasma from all 5 participants on Day 22, suggesting that there was no or negligible systemic uptake of TCP-25 from treated wound areas by the blood.

### Exploratory endpoint

**Fig. 3:**
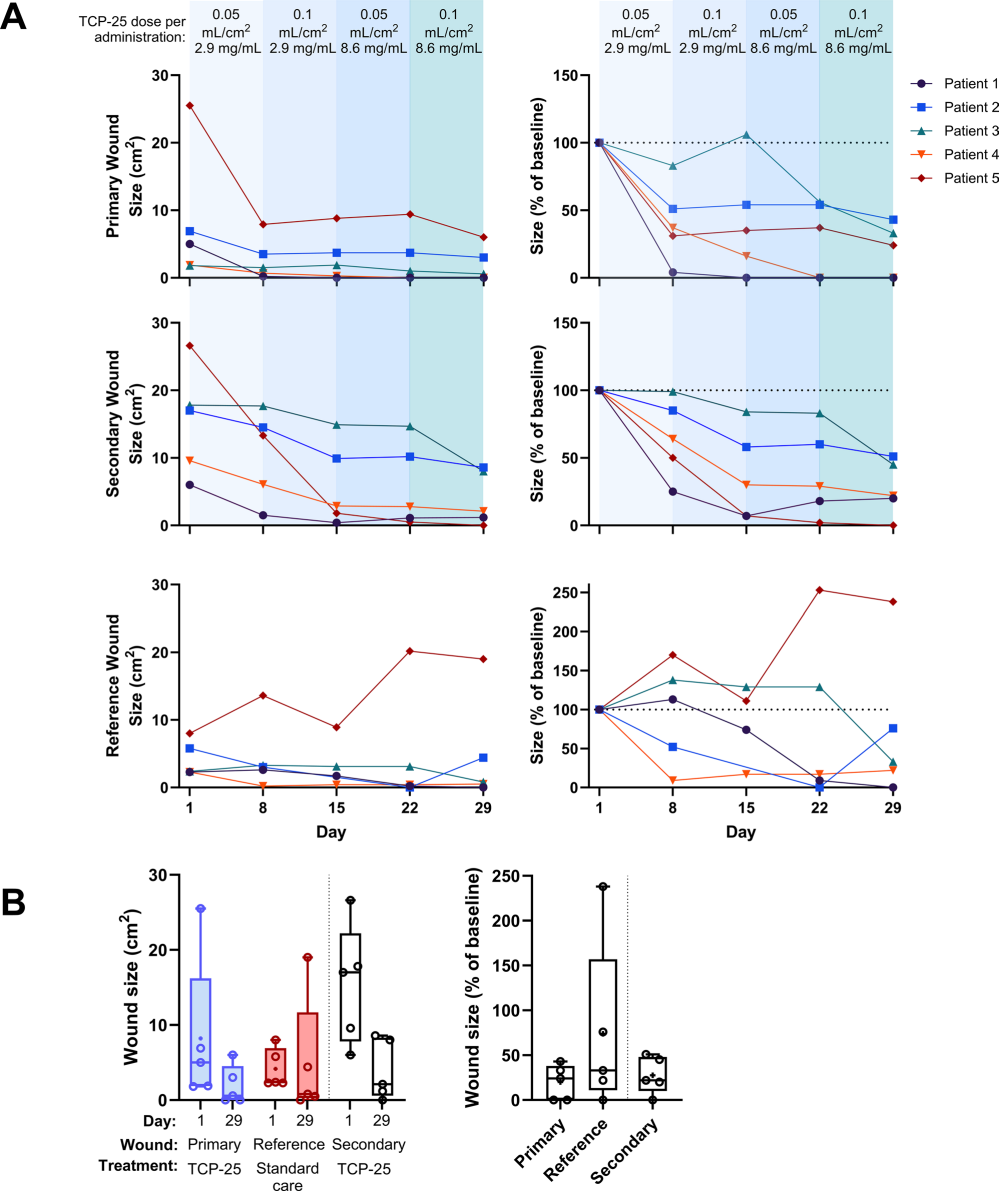
**A**) Open wound size in cm^2^ and percent of baseline over time in primary, secondary, and reference wounds. **B**) Open wound size in cm^2^ on Day 1 vs Day 29 (left) and percent of baseline on Day 29 (right). Data shown are the median (line), interquartile range (boxes), and range (whiskers). Each point represents 1 wound. 1 data point missing for patient 2 on day 15, reference wound

Open wound size varied between participants and between primary, secondary, and reference wounds at baseline and over time (Fig. 3). Primary wounds at baseline (Day 1, Visit 2) had a median open wound size of 5.0 cm^2^ (range 1.8–25.5 cm^2^). Following the start of application of TCP-25 gel, the open wound size of all primary wounds decreased over time, wherein 3 of 5 wounds achieved at least 50% wound closure by Day 8 (Fig. 3A). By Day 29, 2 of the primary wounds had closed completely, and all 5 wounds had at least 50% wound closure relative to their size at baseline. Between baseline and Day 29, the open wound size of the primary wounds declined by a median of 3.9 cm^2^ (range 1.2–19.5 cm^2^), corresponding to a median percent area reduction of 76% (range 57–100%).

Reference wounds, which had a median open wound area of 2.4 cm^2^ (range 2.3–8.0 cm^2^) at baseline, were not treated with TCP-25 (Fig. 3A). Unlike TCP-25-treated wounds, the reference wounds did not decrease in size consistently during the study period. Three of the 5 reference wounds increased in open wound area on Day 8, 2 of which remained above their baseline size until at least Day 22. This finding contrasts sharply with the TCP-25-treated wounds, the areas of all of which fell after 1 week of treatment and continued to decline steadily thereafter. By Day 29, only 1 of the 5 reference wounds had closed completely, and only 3 had experienced at least 50% wound closure. On Day 29, the reference wounds had decreased by 1.6 cm^2^ (range decrease of 2.3 to an increase of 11.0 cm^2^), corresponding to a median percent area reduction of 67% (range decrease of 100% to an increase of 138%).

Secondary wounds, which were larger and more complex due to their location and chronicity compared with primary wounds, had a median open wound area of 17.0 cm^2^ (range 6.0–26.6 cm^2^) at baseline (Fig. 3A). Despite their larger size and complexity, most secondary wounds also declined steadily in size following the start of application of TCP-25 gel. By Day 29, 4 of the 5 secondary wounds had over 50% wound closure, and 1 of the wounds had closed completely. The secondary wounds underwent a median decrease of 8.4 cm^2^ (range 4.8–26.6 cm^2^) and a median percent area reduction of 78% (range 49–100%) on Day 29 relative to their size at baseline.

Taken together, as summarized in Fig. 3B, treatment with TCP-25 gel led to decreases in open wound size, wherein primary wounds showed a median percent area reduction of 76% by Day 29 and consistent healing, including complete closure in 2 cases, whereas untreated reference wounds exhibited inconsistent changes, with some increasing in size and only 1 achieving complete closure. Secondary wounds, which were larger and more complex, also responded well to TCP-25 gel, with a median percent area decline of 78% by Day 29, including complete closure in 1 case and over 50% closure in 4 of the 5 wounds. Notably, the trend of improved healing in TCP-25-treated wounds was already apparent after just 1 week (Supplementary Figure 1). All wound size data reported in Fig. 3 and related statistical testing are presented in Supplementary Table 5 and 6.

## Discussion

This study constitutes the first clinical trial to test the administration of TCP-25 in patients with EB with regard to its safety, tolerability, and systemic exposure. TCP-25 gel was administered topically in 2 strengths (2.9 mg/mL and 8.6 mg/mL) across 12 doses and found to be safe and well tolerated, based on AEs and local tolerability evaluations. Further, we did not observe any systemic uptake of TCP-25. This small study also provided initial indications of its efficacy: wounds that were treated with TCP-25 appeared to undergo more rapid initial wound healing, based on observed wound area measurements and wound status, and had smaller open wound areas at the end of the study compared with untreated reference wounds.

We found TCP-25 to be safe and well tolerated in our cohort of 5 RDEB patients. No deaths, SAEs, or other significant AEs occurred as a result of treatment with TCP-25, and no participant withdrew due to AEs at any stage of the study. Three of the 5 participants experienced wound-associated AEs of mild to moderate intensity, one of which was possibly related to the treatment and likely due to gel adhesion. Additionally, no abnormal local reactions were reported in any wound treated with TCP-25, nor in any reference wound, compared to expected wound healing outcomes. These findings are consistent with previous experimental studies that administered TCP-25 in murine and porcine models and did not detect any AEs [29, 38]. The fact that endogenous TCP sequences identical to synthetically produced TCP-25 are present in human wound fluid minimizes the risk of adverse reactions [27, 28].

No participant experienced systemic uptake of TCP-25. However, only 1 measurement of plasma TCP-25 was taken, following the first administration of the highest dose level, which limits the pharmacokinetic assessment. This was due to ethical concerns regarding repeated venipuncture in DEB patients with fragile skin, and in accordance with regulatory authority recommendations. However, current results still align with previous preclinical findings, which indicated no uptake of TCP-25 2 and 24 hours after topical application to porcine skin and partial thickness wounds [29]. These data are also consistent with the molecular properties of TCP-25 and the penetration of skin and wounds by similar peptides. In general, peptides undergo little to no penetration of the skin [39], and charged peptides above 1 kDa have poor penetration across the skin barrier [40]. TCP-25 is positively charged and has a molecular weight above 3 kDa. Although open wounds lack an epidermal barrier, fewer than 1% of peptides from an open wound are expected to undergo systemic uptake [41]. Other cationic peptide drugs, such as Regranex, experience low or undetectable systemic absorption when applied topically to wounds in humans [42]. Thus, the lack of any significant systemic uptake of TCP-25 in our study corresponds with its expected behavior.

In this study, TCP-25 was applied to a relatively large area. Each patient had two 50-cm^2^ wound treatment areas which received TCP-25 (i.e., 100 cm^2^ in total). This included an open wound and the surrounding periwound area, the largest of which had a total open wound size (the sum of the areas of the primary and secondary wounds) at baseline of 52.1 cm^2^. RDEB tends to cause many wounds over the entire body; thus, the area over which TCP-25 is applied in actuality—encompassing more open wound areas—is likely to be much larger than those that were treated in this study. Given the poor absorption of similar peptide drugs, it is unlikely that TCP-25 would be absorbed systemically, even with a large application area; however, this aspect should be confirmed in future studies.

There were indications that TCP-25 had efficacy in these participants with regard to wound healing. Although the sample size of the study was small and not sufficiently powered to detect differences in wound size, we noted possible effects of TCP-25 on wound healing dynamics, based on visualization of the wounds and measurement of their area over time. Notably, primary wounds and more complicated secondary wounds began to decline in size already 1 week after administration of TCP-25, and most TCP-25-treated wounds continued to decrease steadily until the end of the treatment period. Two of the 5 primary wounds had closed completely by Day 29, and all were less than 50% of their initial size. Wounds in DEB patients are difficult to heal and dynamic in nature. An earlier study reported a mean time to wound closure of 49.4 days in 10–50-cm^2^ placebo-treated DEB wounds [43]. Thus, a treatment that accelerates wound healing could significantly improve outcomes for EB patients.

However, there are few alternatives for medical treatment of this disease. Current management of inherited EB essentially consists of avoiding traumas that trigger lesions, preventing infection, and facilitating healing of the wounds with systematic use of bandages and dressings [21, 44]. Further, existing treatments are often associated with negative effects. Applied dressings must be changed frequently until the wounds heal, and the accompanying manipulation of the skin risks the creation of additional wounds [21]. Infected and heavily colonized wounds are treated with mild antiseptics, such as chlorhexidine, polyhexanide [polyhexamethylene biguanide (PHMB)], and dilute solutions of sodium hypochlorite, potassium permanganate or acetic acid. However, PHMB generally does not benefit wound infections [45, 46], and safety concerns have been raised over its potential carcinogenicity [25]. Topical antimicrobials are recommended for critically infected or colonized wounds, whereas systemic antibiotics can be used to treat multiple infected lesions. However, in general topical antibiotics have not demonstrated improved wound healing outcomes and can often cause patient discomfort and adverse reactions [24, 47].

Infection management in EB is complicated by the waning efficacy of antimicrobial agents and antiseptics due to increasing antimicrobial resistance. Topical antibiotic use must be restricted to short periods, because prolonged application increases the risk of antimicrobial resistance and sensitization of the patient [21] . Antimicrobial-resistant bacteria are often present in EB wounds, likely as a consequence of the frequent use of antibiotics in these patients, thus increasing the risk of antimicrobial-resistant infections [17, 18]. It is generally believed, supported by earlier studies [48, 49], that bacterial resistance to antimicrobial peptides is harder to develop than resistance to antibiotics. However, potential microbial resistance towards TCP-25 should be directly addressed in future long-term preclinical and clinical studies.

With regard to its mechanism of action, TCP-25 reduces inflammation while simultaneously controlling bacteria and their proinflammatory products, improving wound healing [29]. This dual mechanism is important for wound healing in general and especially for EB wounds. In EB, excess, chronic, and imbalanced inflammation impairs wound healing, elicits further tissue damage, and promotes pain and itching [1]. Inflammation in DEB wounds is also associated with the development of fibrosis, which causes debilitating deformities and scarring, and encourages the development of squamous cell carcinoma [50]. The abnormal inflammation is exacerbated by the presence of microbes and an altered microbiome, including the presence of potentially pathogenic species *Staphylococcus aureus* and *Pseudomonas aeruginosa*, which in turn increase the risk of infection [14, 15–19]. *Candida* species are also frequently found in wound cultures in EB. [15] The TLR signaling and subsequent inflammation that is activated by these microbes delays wound healing [20], which in turn leads to the extended presence of open wounds and areas of denuded skin and the loss of the stratum corneum barrier [6]. These effects heighten the risk for systemic bacterial infections and sepsis [5].

Consequently, to heal wounds in EB efficiently, this spiral involving inflammation and infection must be prevented and treated, and in particular, the excessive component of the ensuing inflammation needs to be addressed. Local gene therapies such as Vyjuvek and the recently approved autologous gene-modified keratinocyte treatment Zevaskyn (prademagene zamikeracel) target the underlying genetic cause and have shown great promise in clinical trials [51, 52]. However, significant challenges persist. Even with these advanced therapies, not all treated wounds heal completely. Additional limitations include constraints on treatable wound size, concerns about immunogenicity, and largely unknown long-term effects. Thus, additional complementary strategies that facilitate wound healing by controlling inflammation and infection are still needed to improve EB symptoms and mitigate the severe consequences of EB wounds.

The strengths of this study include the fact that it is the first testing of a relevant and promising new treatment modality in EB patients that is based on natural peptide-based host defense mechanisms. Further, our trial implemented a sound internal control, wherein each participant bore 2 TCP-25-treated wounds and a reference wound. Additionally, our inclusion of more complicated wounds, which often contribute significantly to morbidity, provided valuable insights into the effects of TCP-25 on varying wound types.

Several study limitations, however, prevent generalization of the results. In addition to the small sample size, this trial was not randomized or blinded. Additionally, only RDEB patients were included, which limits the ability to generalize wound healing results to the dominant DEB population, which typically experiences less severe wounds. The treated area was limited, relative to the area that may be required in certain EB patients. The fact that the second and third applications of the gel were conducted at home, outside of the controlled clinical setting, and the fact that compliance verification relied on the return of empty tubes both potentially limited our control of compliance with the application procedure. This could have caused differences in application of TCP-25 gel between patients in addition to those recorded as protocol deviations, which could have influenced the results of both the safety and exploratory efficacy assessments in the TCP-25 treated wounds. No efficacy evaluation of possible antibacterial effects was conducted due to limitations in the study design, such as the lack of a proper run-in period to establish baseline values and the limited sample size. Finally, our treatment time was narrow, preventing us from determining the longevity and long-term efficacy of TCP-25, given that EB wounds typically take longer to heal, requiring TCP-25 to be applied for much longer durations.

## Conclusions

Topical TCP-25 gel was safe and well tolerated by the study participants with RDEB, and we did not observe any systemic uptake of TCP-25 in them. The dual anti-inflammatory and antimicrobial activity of TCP-25 could be highly beneficial in EB, which is driven by dysfunctional inflammation and dysbiosis. We noted a possible positive treatment effect of TCP-25 on wound healing speed and open wound area reduction. To this end, a larger study that is powered for detecting efficacy with regard to wound healing parameters is planned and will further examine this promising new concept for wound healing.

## Electronic supplementary material

Below is the link to the electronic supplementary material.

Supplementary Material 1

## Data Availability

The data that support the findings of this study are available from the corresponding author upon reasonable request, with the exception of information that cannot be shared publicly for formal reasons as it could compromise the privacy of research participants.
