# Supplementary material for: Results of a non-randomized, open-label phase I study evaluating the novel immunomodulatory peptide TCP-25 for treatment of dystrophic epidermolysis bullosa
Source: Orphanet J Rare Dis. 2025 Dec 2;21:4. doi: 10.1186/s13023-025-04156-7 (PMC12777436; doi:10.1186/s13023-025-04156-7)
Supplement: Supplementary file 1 — Supplementary Material 1 [file 13023_2025_4156_MOESM1_ESM.docx]

**Supplementary Material for “Results of a Non-Randomized, Open-Label Phase I Study Evaluating the Novel Immunomodulatory Peptide TCP-25 for Treatment of Dystrophic Epidermolysis Bullosa”**

Contents

[Detailed inclusion and exclusion criteria 2](#_Toc207807939)

[Definition of causality of AEs 4](#_Toc207807940)

[Concomitant medications 4](#_Toc207807941)

[Supplementary Table 1. 4](#_Toc207807942)

[Treatment compliance and protocol deviations 6](#_Toc207807943)

[Adverse events 7](#_Toc207807944)

[Supplementary Table 2. 7](#_Toc207807945)

[Supplementary Table 3. 8](#_Toc207807946)

[Supplementary Table 4. 9](#_Toc207807947)

[Open wound size 10](#_Toc207807948)

[Supplementary Figure 1 10](#_Toc207807949)

[Supplementary Table 5. 11](#_Toc207807950)

[Supplementary Table 6. 12](#_Toc207807951)

# Detailed inclusion and exclusion criteria

The detailed inclusion and exclusion criteria are as follows:

Inclusion criteria:

1. Willing and able to give written informed consent for participation in the study. For 15 to 17-year-olds: A separate consent was required from both (if applicable) the patient’s parents/legal guardians.
2. Male or female patient with a documented diagnosis of inherited DEB, ≥15 years of age at the time of signing the informed consent.
3. Male patients had to be willing to use condom or be vasectomised or practice sexual abstinence to prevent pregnancy and drug exposure of a partner and refrain from donating sperm from the date of the last dosing until 3 months after the last dosing with the IMP. Their female partner of childbearing potential had to use contraceptive methods with a failure rate of < 1% to prevent pregnancy.
4. WOCBP had to practice abstinence (only allowed when this was the preferred and usual lifestyle of the patient) or had to agree to use a highly effective method of contraception with a failure rate of < 1% to prevent pregnancy (combined [oestrogen and progestogen containing] hormonal contraception associated with inhibition of ovulation [oral, intravaginal, transdermal], progestogen-only hormonal contraception associated with inhibition of ovulation [oral, injectable, implantable], IUD or IUS) from at least 4 weeks prior to dose to 4 weeks after last dose. Female patients had to refrain from donating eggs from the date of dosing until 3 months after dosing with the IMP. Their male partner had to agree to use a condom during the same time frame if he had not undergone vasectomy.
5. Women of non-childbearing potential were defined as pre-menopausal females who were sterilised (tubal ligation or permanent bilateral occlusion of fallopian tubes); or females who had undergone hysterectomy or bilateral oophorectomy; or post-menopausal defined as 12 months of amenorrhea (in questionable cases a blood sample with detection of FSH 25-140 IU/L was confirmatory).
6. Clinically relevant medical history at the time of screening, as judged by the Investigator.
7. Presence of 2 target wound areas of 50 cm2. The primary wound area was not to be located at anatomical sites with high likelihood of accidental trauma (e.g., knee, elbow). The secondary wound area could be located at anatomical sites with high likelihood of accidental trauma or be within the higher age span. Both wound areas had to meet all the following characteristics:
   1. Including open wound with a surface area of ≤ 30 cm2 (as measured with the Silhouette imaging equipment at the screening visit).
   2. At Visit 2 (first IMP treatment), each target wound was not to present a surface reduction ≥ 30 % from the Screening visit.
   3. Wound aged ≥ 3 weeks to < 9 months at the Screening visit.
8. Presence of a reference wound area of 50 cm2 to be treated with standard of care only and to be included in the exploratory assessment. The wound area had to match the primary wound area for the following characteristics:
   1. Including open wound with a surface area of ≤ 30 cm2 (as measured with the Silhouette imaging equipment at the screening visit).
   2. At Visit 2 (first IMP treatment), the wound was not to present a surface reduction ≥ 30 % from the Screening visit.
   3. Wound aged ≥ 3 weeks to < 9 months at the Screening visit.
   4. Not located at anatomical sites with high likelihood of accidental trauma (e.g., knee, elbow).
9. Willing to attend study site visits.

Exclusion criteria

Patients could not enter the study if any of the following exclusion criteria were fulfilled:

1. Any subtype of EB other than DEB.

2. EB index wounds that had an infection in need of systemic antibiotic treatment.

3. Presence of, or documented illness, which in the Investigator’s opinion could negatively affect wound healing or interfere with the study conduct.

4. Subject had evidence of a systemic infection or had used systemic antibiotics for EB related infections within 7 days before the screening visit (Visit 1).

5. Administration of systemic corticosteroids within 30 days (> 10 mg daily of prednisolone or corresponding) or topical corticosteroids on target wound areas (primary, secondary and reference wound areas) within 14 days before the screening visit (Visit 1). Corticosteroids for inhalation, ophthalmic, or intranasal use were permitted.

6. Subject had undergone stem cell transplant or gene therapy for the treatment of EB with an effect on target wounds.

7. History of malignancy, including basal cell carcinomas or squamous cell carcinomas in the wound areas that were to be included in the study.

8. History of severe allergy/hypersensitivity or ongoing allergy/hypersensitivity, as judged by the Investigator, or history of hypersensitivity to drugs with a similar chemical structure or class to TCP-25 or any excipients of the hydrogel.

9. Planned treatment or treatment with another investigational drug within 3 months prior to Day -1.

10. Involvement in the planning and/or conduct of the study.

11. Investigator considered the patient unlikely to comply with study procedures, restrictions, and requirements.

# Definition of causality of AEs

The causal relationship with the treatment was defined as follows:

| **Probable** | The event has a strong temporal relationship to the TCP-25 gel/procedure or recurs on re-challenge, and another etiology is unlikely or significantly less likely. |
| --- | --- |
| **Possible** | The event has a suggestive temporal relationship to the TCP-25 gel/procedure, and an alternative etiology is equally or less likely. |
| **Unlikely** | The event has no temporal relationship to the TCP-25 gel/procedure or is due to underlying/concurrent illness or effect of another drug (that is, there is no causal relationship between the TCP-25 gel/procedure and the event). |

# Concomitant medications

Supplementary Table 1. Concomitant medications by WHODrug preferred name.

|  | Total (N=5) | |
| --- | --- | --- |
| Preferred name | n (%) | m |
| **Total** | **5 (100%)** | **78** |
| Alimemazine tartrate | 4 (80%) | 4 |
| Betamethasone valerate | 3 (60%) | 4 |
| Betamethasone;Neomycin | 3 (60%) | 3 |
| Bromhexine hydrochloride;Ephedrine hydrochloride | 1 (20%) | 1 |
| Calcium carbonate;Colecalciferol | 1 (20%) | 1 |
| Carbohydrates nos;Carnitine;Choline;Fats nos;Fibre, dietary;Inositol; Minerals nos;Proteins nos | 1 (20%) | 1 |
| Carbohydrates nos;Fats nos;Minerals nos;Proteins nos;Vitamins nos | 1 (20%) | 1 |
| Carbomer | 4 (80%) | 4 |
| Chloramphenicol | 2 (40%) | 2 |
| Chlorine | 1 (20%) | 1 |
| Ciclosporin | 1 (20%) | 1 |
| Clobetasol propionate | 1 (20%) | 1 |
| Clobetasone butyrate | 1 (20%) | 1 |
| Colecalciferol | 3 (60%) | 3 |
| Doxycycline monohydrate | 1 (20%) | 1 |
| Esomeprazole magnesium | 1 (20%) | 1 |
| Fats nos | 1 (20%) | 1 |
| Fluconazole | 1 (20%) | 1 |
| Folic acid | 1 (20%) | 1 |
| Gabapentin | 2 (40%) | 2 |
| Glycerol | 2 (40%) | 2 |
| Hydrocortisone acetate;Oxytetracycline hydrochloride;Polymyxin b sulfate | 1 (20%) | 1 |
| Hydrocortisone butyrate | 1 (20%) | 1 |
| Hypromellose | 1 (20%) | 1 |
| Ibuprofen | 3 (60%) | 3 |
| Lansoprazole | 1 (20%) | 1 |
| Levocabastine hydrochloride | 1 (20%) | 1 |
| Loperamide hydrochloride | 1 (20%) | 1 |
| Losartan | 1 (20%) | 1 |
| Macrogol | 1 (20%) | 1 |
| Macrogol 3350;Potassium chloride;Sodium bicarbonate;Sodium chloride | 2 (40%) | 2 |
| Magnesium hydroxide | 1 (20%) | 1 |
| Methotrexate | 1 (20%) | 2 |
| Nutrients nos | 1 (20%) | 1 |
| Other emollients and protectives | 1 (20%) | 1 |
| Other ophthalmologicals | 1 (20%) | 1 |
| Paracetamol | 3 (60%) | 4 |
| Paraffin, liquid;White soft paraffin | 2 (40%) | 2 |
| Petrolatum | 1 (20%) | 1 |
| Potassium permanganate | 2 (40%) | 2 |
| Prednisolone | 1 (20%) | 1 |
| Propylene glycol | 3 (60%) | 3 |
| Salbutamol sulfate | 1 (20%) | 1 |
| Zinc products | 3 (60%) | 3 |
| Zinc sulfate | 5 (100%) | 5 |
|  | | |

N: number of patients in treatment group. Percentages are based on N. n: number of patients. M: number of events.

# Treatment compliance and protocol deviations

1. There were 2 deviations affecting 1 participant (participant 5).
   1. The participant used double the planned amount of TCP-25 gel (8.6 mg/ml; 0.1 mL/cm^2^, instead of 0.05 mL/cm^2^) on Day 17.
   2. The participant then used half of the planned amount of TCP-25 gel (8.6 mg/ml; 0.05 mL/cm^2^, instead of 0.1 mL/cm^2^) on Day 24.
2. Two participants (participants 2 and 3) used only 1 vial/wound (2.9 mg/mL) on Day 10 and Day 13.

# Adverse events

Supplementary Table 2. Overview of adverse events

|  | Treatment period 1 0.145 05 mL/cm^2^  TCP-25 2.9 mg/cm^2^ mL (N=5) | | Treatment period 2 0.1 mL/cm^2^  TCP-25 2.9 mg/mL (N=5) | | Treatment period 3  0.05 mL/cm^2^  TCP-25 8.6 mg/mL (N=5) | | Treatment period 4  0.1 mL/cm^2^  TCP-25 8.6 mg/mL  (N=5) | | Total (N=5) | |
| --- | --- | --- | --- | --- | --- | --- | --- | --- | --- | --- |
|  | n (%) | m | n (%) | m | n (%) | m | n (%) | m | n (%) | m |
| **Any AE** | 2 (40%) | 4 | 1 (20%) | 2 | 2 (40%) | 3 | 2 (40%) | 2 | 5 (100%) | 11 |
| **Any SAE** | 0 | 0 | 0 | 0 | 0 | 0 | 0 | 0 | 0 | 0 |
| **Any AE leading to withdrawal from study** | 0 | 0 | 0 | 0 | 0 | 0 | 0 | 0 | 0 | 0 |
| **Any AE leading to death** | 0 | 0 | 0 | 0 | 0 | 0 | 0 | 0 | 0 | 0 |
| **Causality** |  |  |  |  |  |  |  |  |  |  |
| Unlikely Related | 2 (40%) | 4 | 1 (20%) | 2 | 2 (40%) | 2 | 2 (40%) | 2 | 5 (100%) | 10 |
| Possibly Related | 0 | 0 | 0 | 0 | 1 (20%) | 1 | 0 | 0 | 1 (20%) | 1 |
| Probably Related | 0 | 0 | 0 | 0 | 0 | 0 | 0 | 0 | 0 | 0 |
| **Severity** |  |  |  |  |  |  |  |  |  |  |
| Mild | 2 (40%) | 3 | 1 (20%) | 2 | 1 (20%) | 2 | 2 (40%) | 2 | 5 (100%) | 9 |
| Moderate | 1 (20%) | 1 | 0 | 0 | 1 (20%) | 1 | 0 | 0 | 2 (40%) | 2 |
| Severe | 0 | 0 | 0 | 0 | 0 | 0 | 0 | 0 | 0 | 0 |
| Life-Threatening | 0 | 0 | 0 | 0 | 0 | 0 | 0 | 0 | 0 | 0 |
| Death | 0 | 0 | 0 | 0 | 0 | 0 | 0 | 0 | 0 | 0 |
|  | | | | | | | | | | |

N: number of patients in treatment group. Percentages are based on N. n: number of patients. M: number of events.

Supplementary Table 3. Adverse events by system organ class and preferred term

|  | Treatment period 1 0.05 mL/cm^2^ TCP-25 2.9 mg/mL (N=5) | | Treatment period 2 0.1 mL/cm^2^ TCP-25 2.9 mg/mL (N=5) | | Treatment period 3 0.05 mL/cm^2^ TCP-25 8.6 mg/mL (N=5) | | Treatment period 4 0.1 mL/cm^2^ TCP-25 8.6 mg/mL (N=5) | | Total (N=5) | |
| --- | --- | --- | --- | --- | --- | --- | --- | --- | --- | --- |
| System organ class  Preferred term | n (%) | m | n (%) | m | n (%) | m | n (%) | m | n (%) | m |
| **Total** | **2 (40%)** | **4** | **1 (20%)** | **2** | **2 (40%)** | **3** | **2 (40%)** | **2** | **5 (100%)** | **11** |
| **General disorders and administration site conditions** | **1 (20%)** | **1** | **1 (20%)** | **1** | **1 (20%)** | **1** | **0** | **0** | **3 (60%)** | **3** |
| Application site erosion | 0 | 0 | 0 | 0 | 1 (20%) | 1 | 0 | 0 | 1 (20%) | 1 |
| Application site pruritus | 0 | 0 | 1 (20%) | 1 | 0 | 0 | 0 | 0 | 1 (20%) | 1 |
| Pyrexia | 1 (20%) | 1 | 0 | 0 | 0 | 0 | 0 | 0 | 1 (20%) | 1 |
| **Infections and infestations** | **1 (20%)** | **1** | **1 (20%)** | **1** | **0** | **0** | **1 (20%)** | **1** | **3 (60%)** | **3** |
| Nasopharyngitis | 1 (20%) | 1 | 1 (20%) | 1 | 0 | 0 | 1 (20%) | 1 | 3 (60%) | 3 |
| **Injury, poisoning and procedural complications** | **1 (20%)** | **2** | **0** | **0** | **1 (20%)** | **1** | **1 (20%)** | **1** | **2 (40%)** | **4** |
| Wound complication | 1 (20%) | 2 | 0 | 0 | 1 (20%) | 1 | 1 (20%) | 1 | 2 (40%) | 4 |
| **Product issues** | **0** | **0** | **0** | **0** | **1 (20%)** | **1** | **0** | **0** | **1 (20%)** | **1** |
| Product adhesion issue | 0 | 0 | 0 | 0 | 1 (20%) | 1 | 0 | 0 | 1 (20%) | 1 |
|  | | | | | | | | | | |

N: number of patients in treatment group. Percentages are based on N. n: number of patients. M: number of events.

Supplementary Table 4. Wound-associated AEs, Part III

| **Participant** | **AE(s), verbatim text** | **AE(s) PT** | **Wounds (treatment)** | **Intensity** | **Causality** | **Start day** | **End day** | **Comment** |
| --- | --- | --- | --- | --- | --- | --- | --- | --- |
| 1 | Experienced an increased stickiness to dressing | Product adhesion issue | Primary wound (TCP-25) | Mild | Possibly related | 17 | Ongoing at study end | Reported as a single AE |
|  |  |  | Secondary wound (TCP-25) |  |  |  |  |  |
|  | Skin tend to adhere to dressing leaving small erosions in the area | Application site erosion | Secondary wound (TCP-25) | Mild | Unlikely related | 17 | Ongoing at study end |  |
| 2 | Itching in the wound where IMP was put on | Wound  complication | Primary wound (TCP-25) | Mild | Unlikely related | 1 | 3 | Reported as 2 AEs (1/wound) |
|  |  |  | Secondary wound (TCP-25) |  |  |  |  |  |
| 5 | Itching around wound (application site) | Application site pruritus | Primary wound (TCP-25) | Mild | Unlikely related | 10 | 17 |  |
|  | Pain in wound 1 | Wound  complication | Primary wound (TCP-25) | Mild | Unlikely related | 30 | 31 |  |
|  | Itching in wound | Wound  complication | Reference wound (standard of care) | Moderate | Unlikely related | 21 | Ongoing at study end |  |

# Open wound size


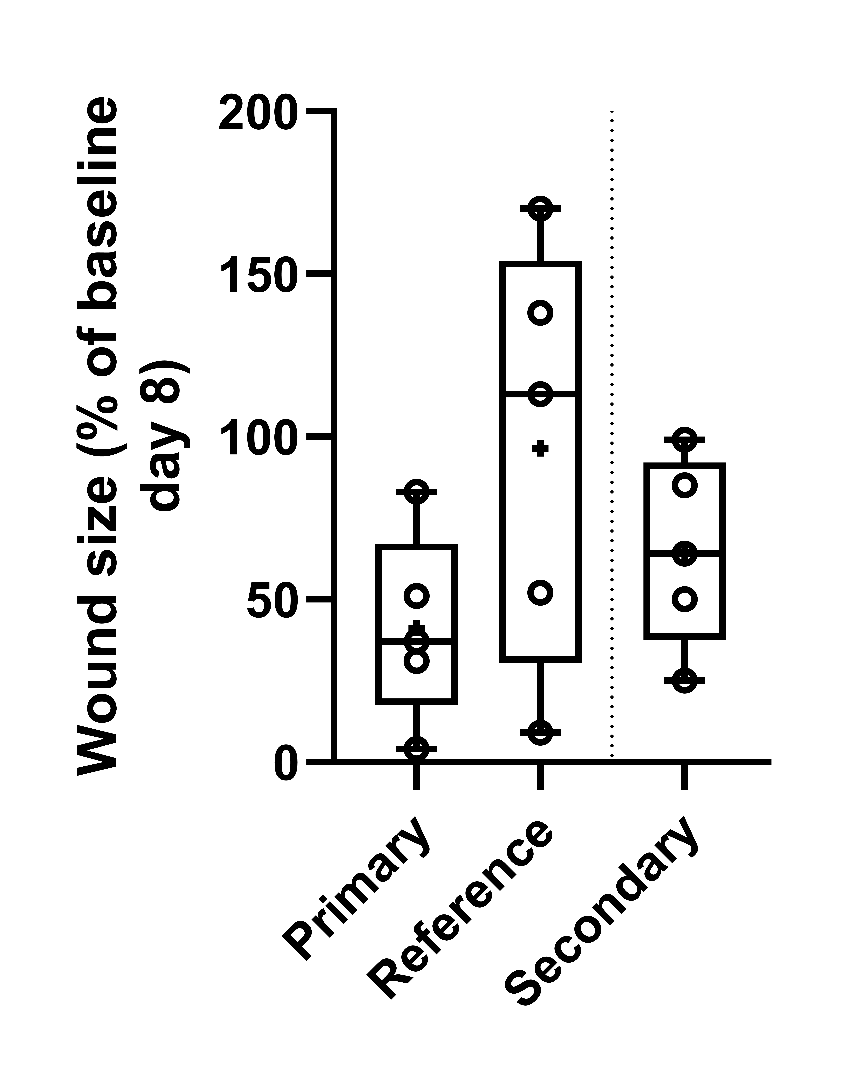


Supplementary Figure 1. Open wound size as a percent of baseline on Day 8. Data shown are the median (line), interquartile range (boxes), and range (whiskers). Each point represents 1 wound. The difference between primary and secondary wound size is not significant (p = 0.152) when analyzed using a paired, two-tailed t-test with no correction for multiple comparisons.

Supplementary Table 5. Open wound area at all time points.

|  |  | **Open wound size (cm^2^)** | | | |  |  |
| --- | --- | --- | --- | --- | --- | --- | --- |
| **Participant** | **Wound** | **Day 1** | **Day 8** | **Day 15** | **Day 22** | **Day 29** | **Complete wound closure Y/N** |
| 1 | Primary wound | 5 | 0.2 | 0 | 0 | 0 | Y |
|  | Reference wound | 2.3 | 2.6 | 1.7 | 0.2 | 0 | Y |
|  | Secondary wound | 6 | 1.5 | 0.4 | 1.1 | 1.2 | N |
| 2 | Primary wound | 6.9 | 3.5 | 3.7 | 3.7 | 3 | N |
|  | Reference wound | 5.8 | 3 | Na* | 0 | 4.4 | N |
|  | Secondary wound | 17 | 14.5 | 9.9 | 10.2 | 8.6 | N |
| 3 | Primary wound | 1.8 | 1.5 | 1.9 | 1 | 0.6 | N |
|  | Reference wound | 2.4 | 3.3 | 3.1 | 3.1 | 0.8 | N |
|  | Secondary wound | 17.8 | 17.7 | 14.9 | 14.7 | 8 | N |
| 4 | Primary wound | 1.9 | 0.7 | 0.3 | 0 | 0 | Y |
|  | Reference wound | 2.3 | 0.2 | 0.4 | 0.4 | 0.5 | N |
|  | Secondary wound | 9.6 | 6.1 | 2.9 | 2.8 | 2.1 | N |
| 5 | Primary wound | 25.5 | 7.9 | 8.8 | 9.4 | 6 | N |
|  | Reference wound | 8 | 13.6 | 8.9 | 20.2 | 19 | N |
|  | Secondary wound | 26.6 | 13.3 | 1.8 | 0.5 | 0 | Y |

* Datapoint missing due to investigator measuring the wrong wound.

Supplementary Table 6. Reporting of exact p-values. Primary, secondary, and reference wounds were compared by the Friedman test with Dunn’s correction for multiple comparisons. P-values were calculated by GraphPad Prism version 10.4.1.

|  | Primary vs reference* | Primary vs secondary | Secondary vs reference |
| --- | --- | --- | --- |
| Wound size day 1 | >0,99 | 0,08 | 0,08 |
| Wound size day 29 | 0,62 | 0,12 | >0,99 |
| Percentage change (baseline vs day 29) | 0,46 | 0,46 | >0,99 |
| Absolute change (baseline vs day 29) | 0,62 | 0,62 | 0,03 |

* Comparison of matched wounds.
